# *Auricularia auricula-judae* Attenuates the Progression of Metabolic Syndrome in High-Fat Diet-Induced Obese Rats: Enzymatic Pre-Digestion Technology Is Superior to Superfine Grinding Method

**DOI:** 10.3390/foods13030406

**Published:** 2024-01-26

**Authors:** Ying Jia, Kun Chen, Menggang Du, Wanzhou Zhao, Yong Chen, Junhong Cheng, Lin Zhao, Jiankang Liu, Jiangang Long

**Affiliations:** 1 Center for Mitochondrial Biology and Medicine, The Key Laboratory of Biomedical Information Engineering of Ministry of Education, School of Life Science and Technology, Xi’an Jiaotong University, Xi’an 710049, China; 2 Shaanxi 38Fule Special Medical Food Co., Ltd., Shangluo 711400, China; 3 The Nanjing Han & Zaenker Cancer Institute (NHZCI), OG Pharmaceuticals, 88 Jiangdong Road, Nanjing 210036, China; 4 School of Health and Life Sciences, University of Health and Rehabilitation Sciences, Qingdao 266071, China

**Keywords:** *Auricularia auricula-judae*, enzymatic pre-digestion, dyslipidemia, fatty acid synthesis and metabolism, metabolic syndrome, gene signaling, liver function

## Abstract

*Auricularia auricula-judae* (AAJ) has been cultivated for food in China for centuries, and is also used as a folk medicine for the regulation of glucose and lipid metabolism. However, there are few studies on the effects of different processing technologies on the therapeutic efficacy of AAJ to date. This study investigated the effectiveness of the AAJ made by using superfine grinding and enzymatic pre-digestion technologies, respectively, in a high-fat diet obese rat model. It was found that oral administrations of two AAJ products significantly alleviated dyslipidemia by decreasing serum lipid levels and restoring liver functions. AAJ products made by using pre-digestion technology have appreciable potential to ameliorate lipid metabolic disorders over other products, possibly due to the higher levels of dietary fiber, crude polysaccharides, and total flavonoids released from AAJ during processing. By analysis of transcriptome sequencing and protein expression, it was clear that starch and sucrose metabolism and glycerolipid metabolism-related factors involved in fatty acid synthesis and metabolism in the liver of obese rats were significantly improved. This study gives further evidence that AAJ significantly ameliorates the progression of glucose and lipid metabolism in obese rats. Moreover, this study demonstrated for the first time that the pre-digestion method may be a better and more efficient processing approach for the improvement of AAJ bioavailability.

## 1. Introduction

Dyslipidemia is not only the main cause of heart diseases such as atherosclerosis, but also a risk factor for diabetes and hypertension. Dyslipidemia is caused by abnormal blood lipid metabolism, usually manifested as higher levels of serum total cholesterol (TC), triglyceride (TG), low-density lipoprotein cholesterol (LDL-C), and lower levels of high-density lipoprotein cholesterol (HDL-C), as well as fatty liver and obesity, etc. [1].

The World Health Organization estimates that a third of cardiovascular diseases (CVD) are closely associated with dyslipidemia [2]. Given these risk factors, it is important that healthcare pay special attention to long-term adherence to appropriate lipid-lowering intervention in a structured manner alongside monitoring.

So far, many natural health products have been investigated for their potential lipid-lowering purposes, such as soy protein, green tea, bee pollen, plant sterols, probiotic yogurt, hawthorn, and garlic, etc. [3,4,5,6,7,8,9]. In comparison to the potential long-term side effects of statins, all these types of food are safe to use with good evidence for lipid regulating effects.

*Auricularia auricula-judae* (AAJ), also commonly known as black wood ear or black fungus, is a nutritionally and medically significant edible mushroom that is very popular and cultivated in many Asian countries, especially in China. The AAJ planted in Zhashui County, Shaanxi Province, is particularly famous. It has benefited from the unique natural environment in Qinba Mountain Area, which is suitable for the growth of AAJ [10]. Over the past few decades, AAJ has attracted extensive attention due to its antioxidative, hypolipidemic, hypoglycemic, antithrombotic, anti-inflammatory, and antitumoral functions [11,12]. These biological functions are attributed to a series of bioactive materials that AAJ contains: for example, complex polysaccharides, amino acids, flavonoids, vitamins, and minerals with extremely low levels of fat. While the flavonoids from AAJ are helpful in maintaining healthy lipid levels in the body [11], the indigestible polysaccharides from AAJ are fermented by microbiota in the colon to generate short-chain fatty acids, such as butyrate—they are subsequently absorbed into the blood circulation and affect sugar and lipid levels in the body [1]. It was also proved that AAJ polysaccharides could inhibit the absorption of external fat and enhance the metabolism of hepatic fat [1].

The common problems in the consumption of AAJ have arisen with two aspects. On the one hand, most of the existing studies focus on the finished products of AAJ after direct picking and drying. Although AAJ has a variety of health effects, the tough texture of its cell wall structure hinders the full release and absorption of functional components. Therefore, it is important to adopt further deep processing pretreatment to AAJ. On the other hand, polysaccharides are composed of a series of covalently linked monosaccharides. The covalent bonds involved in the formation of polysaccharide chains are easily destroyed by a variety of physical, chemical, and biological factors. In addition, the water solubility of most flavonoids is also poor. Therefore, different processing methods would affect the dissolution and release of AAJ polysaccharides and flavonoids [10,11,12]. However, there are few studies on the effects of different processing technologies on the therapeutic efficacy of AAJ to date.

In this paper, we intended to use two different pretreatment methods, i.e., superfine grinding (SG) and enzymatic pre-digestion (PD), to further process AAJ from Zhashui County. Furthermore, the AAJ products manufactured by different technologies were compared in terms of their effectiveness in lipid regulation of dyslipidemia induced by a high-fat diet in rats. SG technology has been employed with functional food to produce fine powders with a much-enlarged surface area, resulting in increased dispersibility and solubility of the dietary fibers and other nutrients. Moreover, the application of mechanical force during SG could lead to changes in the chemical composition profiles of the polysaccharides. As a result, superfine powders of functional foods usually exhibit stronger biological activities than the original ones, which can be observed in soybean [13], mushrooms [14], and Chinese wolfberry [15], etc. The pretreatment of functional foods using hydrolases has also demonstrated its ability to facilitate the digestibility of polysaccharides and protein and, hence, is useful for improving human consumption because food hydrolysates obtained from enzymatic hydrolysis usually have better functional properties such as higher solubility, improved absorption, better gelling properties, and, consequently, better health effects [16].

To conduct this study, the experiments were intended to (a) analyze the basic components of AAJ products manufactured by SG and PD technologies, respectively; (b) establish and validate the effectiveness in a hyperlipidemic rat model and record the animal body weight and food/water intakes after administration of different AAJ products; (c) measure serial indicators resided in serum and liver; (d) examine the liver and adipose tissue pathology; (e) perform transcriptome sequencing analysis; and (f) analyze the mRNA gene and protein expressions of several essential biomarkers involved in fatty acid synthesis and metabolism in the liver.

## 2. Materials and Methods

### 2.1. Materials

The AAJ used in this study were all from Zhashui County, Shangluo City, Shaanxi Province. To obtain the AAJ product manufactured by SG technology, AAJ was first crushed with a multi-functional crusher (JC-FW-400A multi-functional crusher, Juchuang Group; Qingdao, China), followed by subjection to a jet-milling (LQF-100 laboratory jet mill, Xilei Powder equipment; Shanghai, China) process to obtain a superfine powder of AAJ (500 mesh). Then, the AAJ powder was dissolved into water before the experiments. To obtain AAJ product manufactured by PD technology, AAJ was firstly ground to yield superfine powders, followed by subjecting of the powders to a digestion procedure within digesting solution (the mass ratio of AAJ and water: 1:25) containing a patented ratio of chitinase, cellulase, and pectinase. The mixed solution, with an appropriate temperature and pH for the enzymes, was enzymolysised for 2 h, and then followed by homogeneity and sterilization. Jiaogulan tea (Batch number: 20210716) was purchased from Sheng-Tang-Shan Natural Health Care Products Co., Ltd. (Guangxi, China).

### 2.2. Analysis of Basic Components

The contents of the nutrients in the samples, i.e., total protein, total dietary fiber, crude polysaccharides, and total flavonoids, were determined. Total protein was quantified using the Kjeldahl method. Briefly, the total nitrogen (ammonia) in the samples was liberated at a high temperature, then released into a strong acid, and the content was measured after neutralization and titration [17]. Total dietary fiber was measured after the digestion of protein and starch using thermostable α-amylases, proteases, and glucosidases [18]. Crude polysaccharides were quantified by the phenol–sulfuric acid colorimetric method [19]. Total flavonoids were quantified after absorbing the samples with polyamide powder and eluting the mixture successively with benzene and methanol, followed by colorimetric determination [18].

### 2.3. Animal Grouping and Treatments

An equal number of male and female Sprague Dawley rats (200 ± 10 g) were supplied by the Laboratory Animal Center of Nantong University (Nantong, China) with the license number of SCXK (Su) 2019-0001. All animals were kept in plastic cages in a ventilated room under controlled environmental conditions (ambient temperature, 24–25 °C; humidity, 50–60%; 12/12 h light/dark cycle), with free access to a standard laboratory diet and clean tap water for one week before being used for the experiments. The animals were handled with care according to the NIH (National Institutes of Health USA) (2011) Guide for the Care and Use of Laboratory Animals. All laboratory animals were treated according to the national regulations of the usage and welfare of laboratory animals, and they were approved by the Animal Care and Use Committee in the Han & Zaenker Cancer Institute (Nanjing, China) before the animal experiments (Approval No. OGKQSPF/SQ-36).

Eighty rats were used at the beginning of the experiments. Each group contained an equal number of male and female rats. Ten rats were randomly grouped into Group I (control) and fed with a normal control diet with distilled water during the entire experiment (*n* = 10). The other seventy rats were fed with a high-fat diet (HFD) in reference to a previous report: the formula feed of the mixed hyperlipidemia animal model was added, with 20.0% sucrose, 15% lard oil, 1.2% cholesterol, 0.2% sodium cholate, 1.2% calcium hydrogen phosphate, 0.8% stone powder, and 5.0% casein were added into the maintenance diet for at least 2 weeks until the high levels of serum lipid appeared [20]. Their serum lipid indicators were examined, and sixty rats were confirmed to have dyslipidemia. The dyslipidemia rats, randomly divided into six groups (Group II ~ Group VII, *n* = 10), were fed with the HFD for another 2 months but treated with different formulas. Group II rats were given distilled water daily by oral gavage (10 mL/kg), serving as the HFD model. The rats in Group III, IV, and V received a low- (0.32 g/kg, 8.1 mL/kg), medium- (0.64 g/kg, 16.2 mL/kg), or high-dose (1.28 g/kg, 32.4 mL/kg) of AAJ products manufactured by PD technology daily by oral gavage, serving as the L-AAJ, M-AAJ, and H-AAJ groups, respectively. Group VI rats were given the AAJ product manufactured by SG technology daily by oral gavage (1.28 g/kg), serving as the SG-AAJ group. It should be noted that the dose used in the SG-AAJ group was equal to the dose in H-AAJ group in the base of mass. Group VII rats received Jiaogulan (*Gynostemma pentaphyllum*) tea daily by oral gavage (0.72 g/kg), serving as the positive control group [21]. The body weights of the rats in each group were recorded weekly, and the food/water intakes were recorded daily.

### 2.4. Collection of Blood and Tissue Samples

Before withdrawing the blood and tissue samples, the rats were fasted for 12 h. Blood samples were taken from the orbital venous plexus at 0.5, 1, 1.5, and 2 months. Plasma was separated immediately by centrifugation at 4000 rpm for 10 min and then stored at −20 °C. Upon completion of the experiments, all the animals were sacrificed, and their livers, white adipose tissues (from inguinal and epididymal), and brown adipose tissues (BAT) were excised, rinsed using normal saline, weighed, and their indexes were calculated: liver or fat index = weight of wet liver or fat/body weight × 100%. Each tissue was photographed and divided into two parts: one part was immediately frozen in liquid nitrogen for further investigations into related biochemical markers, and the other part was preserved in 10% formalin and embedded with paraffin for histological analyses.

### 2.5. Biochemical Assays

#### 2.5.1. Serum Biochemistry

Serum concentrations of total cholesterol (TC), triglyceride (TG), low-density lipoprotein cholesterol (LDL-C), high-density lipoprotein cholesterol (HDL-C), cholinesterase (ChE), total bilirubin (TBIL), alanine aminotransferase (ALT), aspartate aminotransferase (AST), insulin (INS), and adiponectin (ADP) were determined using commercial assay kits according to the instructions provided by the manufacturer (Nanjing Jiancheng Bioengineering Institute; Nanjing, China). The blood glucose level was determined immediately with a HEA-230 glucometer (Omron; Osaka, Japan).

#### 2.5.2. Liver Biochemistry

The activities of hepatic superoxide dismutase (SOD) and catalase (CAT), and the levels of hepatic malondialdehyde (MDA), total antioxidant capacity (T-AOC), superoxide radicals, hydroxyl radicals, and DPPH antioxidant capacity were determined using commercial assay kits according to the instructions provided by two manufacturers (EnoGene Biotech and Nanjing Jiancheng Bioengineering Institute; Nanjing, China).

### 2.6. Histological Analyses of Liver and Fat Tissues

The embedded livers and fat tissues were sliced at a thickness of 4~5 μm for each slice using a Leica CM1950 cryostat (Leica Microsystems; Nussloch, Germany) and stained with hematoxylin–eosin (HE). Ten random light microscopic fields of each slice were examined under an Olympus light microscope at 200× (Tokyo, Japan). Pathological changes were scored to evaluate the degree of pathological changes in a blind manner: normal (0 scores), minimal (0.5 scores), mild (1 score), moderate (2 scores), and severe (3 scores). Each score for pathological changes in the liver was the sum of the scores of the hepatocyte vacuolar degeneration and the cell nodules. Each score for pathological changes in fat was the sum of the scores of the size reduction in fat droplets and cellular compactness.

### 2.7. Transcriptome Sequencing

Gene expression profile analysis using RNA sequencing was performed as previously described with minor modifications [22]. Briefly, all RNA samples were extracted from the withdrawn liver tissues using Trizol solution according to the instructions of the manufacturer. The concentrations and the absorption peaks were determined by using a Nanodrop spectrophotometer (Thermo Fisher Scientific; Waltham, MA, USA) at OD260/OD280 to ensure good RNA purity; additionally, the RIN value, 28S/18S ratio, the baseline, and 5S peak were examined by using an Agilent 2100 bioanalyzer system (Agilent Technologies; Santa Clara, CA, USA) to ascertain RNA integrity. The mRNA samples were then isolated using Oligo (dT) magnetic beads, which was followed by random fragmentation of mRNA using a fragmentation buffer. Using mRNA as the template, the first cDNA strand was synthesized from a random hexamer; then, the second cDNA strand was synthesized after the addition of buffer, dNTPs, RNase H, and DNA polymerase. cDNA was purified by AMPure XP beads, which were then subjected into terminal repair and adaptor ligation. The cDNA library was finally constructed by polymerase chain reaction (PCR) amplification. Before online sequencing, the cDNA library was examined by two-step quantifications. Firstly, preliminary determination of nucleic acids’ contents in the cDNA library was performed using a Qubit 2.0 Fluorometer (Thermo Fisher Scientific) with the insert size detected by using an Agilent 2100 bioanalyzer system. Then, accurate quantification of the final concentration of the nucleic acids in the cDNA library was conducted by using Q-PCR (LOQ > 2 nM). cDNA pooling was performed using qualified libraries, and the samples were sequenced (PE 150 reads) by using a NovaSeq 6000 platform (Illumina; San Diego, CA, USA). The raw data acquired were filtered to obtain high-quality clean data, followed by comparing the latter with the reference genome to obtain mapped data. The quality of the sequencing library was evaluated by examining the length of the insert fragment and measuring the random sequences, and followed by performing expression analysis, alternative splicing analysis, new gene mining, gene structure optimizations, etc. Then, analyses such as differential expression, gene function annotation, and enrichment were performed based on the expression levels of genes in different samples or different sample groups.

### 2.8. Real-Time Reverse Transcription PCR Analysis

Total RNA from liver tissue samples was isolated using TRIpure Reagent (Aidlab; Beijing, China) and reverse-transcribed to obtain cDNA using a OneScript™ cDNA Synthesis Kit (Applied Biological Materials; Richmond, BC, Canada). The mRNA expression of several key factors in fatty acid synthesis and metabolism, i.e., Akr1b10, Gpx1, Srebfl, Fasn, Acly, Cyp27a1, Pnpla3, PPARg, Plaat3, and Lpin1, were detected by using real-time PCR using specific primers for each gene. The primer sequence was listed in Table 1. EvaGreen Express 2× qPCR and MasterMix-ROX were used according to the manufacturer’s instructions (Applied Biological Materials). Samples were denatured at 95 °C for 3 min, followed by 40 cycles of 95 °C for 5 s and 60 °C for 15 s. The relative RNA levels were determined using the 2^−ΔΔCt^ method.

### 2.9. Western Blot Analysis

Liver tissues (30 mg for each sample) were lysed in RIPA buffer (EnoGene Biotech) containing freshly added protease/phosphatase inhibitors and PMSF by using a cryo-milling system (Jingxin Experimental Technology; Shanghai, China) and centrifuged at 4 °C for 15 min using an 5415R refrigerated centrifuge (Eppendorf; Hamburg, Germany). The supernatant was collected and the protein levels were determined using a BCA protein assay kit (EnoGene Biotech). Lysates were resolved by employing the sodium dodecyl sulfate–polyacrylamide gel electrophoresis method (SDS-PAGE) and then transferred to polyvinylidene fluoride (PVDF) membrane (Millipore; Billerica, MA, USA). The membrane was then soaked in blocking solution, i.e., 3% bovine albumin serum (BSA) in Tris-buffered saline with 0.1% Tween 20 (TBST), for 1 h, followed by incubation with corresponding primary antibodies at room temperature for 1 h. The membranes were rinsed with TBST solution 4 times, incubated in 1% BSA-TBST solution containing HRP conjugated secondary antibody at room temperature for 1 h, and then washed with TBST solution 4 times again. The chemiluminescence signals were detected with a Tanon 4200 imaging system (Tanon Technology; Shanghai, China) using an ECL detection kit (EnoGene Biotech). The antibodies for Akr1b10 (E97823), Gpx1 (E91110), Srebfl (E2620152), Fasn (E90461), Cyp27a1 (E91982), Pnpla3 (E2516694), PPARg (E90270), and Lpin1(E98486) were purchased from EnoGene. The GAPDH polyclonal antibody and HRP-conjugated goat anti-rabbit IgG secondary antibody were obtained from the same manufacturer. Densitometric analysis of the bands relative to GAPDH internal control was performed using Image J software (version 1.8.0; National Institutes of Health, Bethesda, MD, USA).

### 2.10. Statistical Analysis

Data are presented as means ± SD. The *t*-test or one-way ANOVA (Dunnett) test was performed using GraphPad Prism 5.0. Differences were considered significant when *p* < 0.05 or *p* < 0.01.

## 3. Results

### 3.1. Analysis of the Basic Components

The basic components of AAJ products made by using PD and SG technologies are shown in Table 2. It was found that the contents of dietary fiber, crude polysaccharides, and total flavonoids in the AAJ product made by using PD were much higher than those in the AAJ product made by using SG technology. In particular, total flavonoids are nearly 50 times higher in PD product over SG product.

### 3.2. Body Weight and Food/Water Intakes

As shown in Table 3, significant increases in the body weight of the rats in the HFD group (*p* < 0.05) were recorded compared to the control group after 2 weeks, suggesting that the HFD had successfully induced obesity. Then, after another 2 weeks (week 4), the rats in the SG-AAJ, M-AAJ, H-AAJ, and positive control groups generally showed significantly lower (*p* < 0.05) body weights compared with those of the HFD group. From week 5, the body weight in the SG-AAJ, M-AAJ, H-AAJ, and positive control groups were even lower compared to the HFD group (*p* < 0.01). Though the L-AAJ group condition was not as effective as the higher doses (M-AAJ and H-AAJ) in reducing the body weight, significantly reduced body weights were recorded in the L-AAJ group from week 7 (*p* < 0.05) compared with the HFD group. The effects on controlling the body weight followed the sequence of H-AAJ > M-AAJ > SG-AAJ > L-AAJ. It was also interesting to notice that both the SG-AAJ group and H-AAJ group presented a clear effect in reducing body weight, and the body weights in these two groups did not show significant differences in each week. The fluctuations in food and water intakes were large in all groups during the experiments; however, it was still observed that there was a significant difference (*p* < 0.05) in food intakes between the H-AAJ (84.5 ± 38.0 g) and HFD groups (120.1 ± 3.7 g) on the final day of the experiment.

### 3.3. Serum Biochemistry

**Figure 1:**
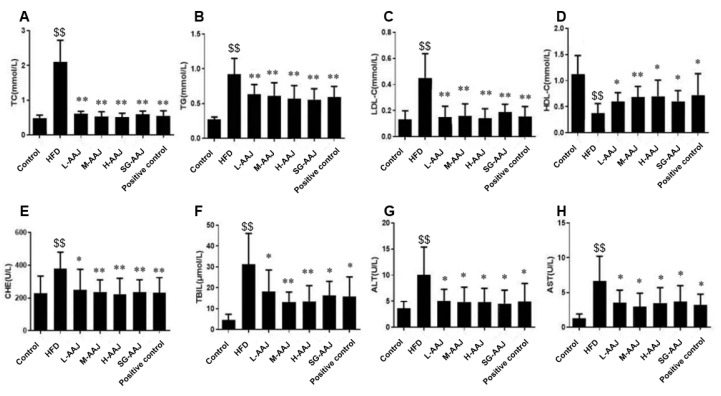
Effects of AAJ interventions on the serum lipid (**A**–**D**) and hepatic function indicators (**E**–**H**). The levels of TC (mmol/L) (**A**), TG (mmol/L) (**B**), LDL-C (mmol/L) (**C**), HDL-C (mmol/L) (**D**), ChE (U/L) (**E**), TBIL (μmol/L) (**F**), ALT (U/L) (**G**), and AST (U/L) (**H**). The results are expressed as mean ± standard deviation (SD, *n* = 10). Statistical analysis was performed using ANOVA. The different superscripts indicate significant differences. ^$$^ *p* < 0.01, HFD vs. control; * *p* < 0.05, ** *p* < 0.01, HFD vs. other groups.

After undergoing the HFD for 2 weeks, TC, TG, and LDL-C levels in the serum were significantly higher than those in the control group (*p* < 0.05), and the HDL-C level was significantly lower (*p* < 0.05), suggesting that the dyslipidemia model was successfully established in rats. After providing AAJ products for 2 weeks, significant decreases (*p* < 0.01) in the serum TC, TG, and LDL-C levels were observed in all the AAJ treatment groups and the positive control group compared with those in the HFD group. For example, after giving AAJ product for 2 weeks, the levels of TC, TG, and LDL-C in the L-AAJ group were 0.69 ± 0.20, 0.62 ± 0.19, and 0.23 ± 0.10 mmol/L, respectively, which were significantly lower than the counterparts in the HFD group (2.26 ± 0.50, 0.93 ± 0.17 and 0.46 ± 0.17 mmol/L, respectively). Similarly, after giving AAJ product for 2 months, the levels of TC, TG, and LDL-C in the L-AAJ group were still significantly lower than in the counterparts in the HFD group, which is shown in Figure 1A–C. In the meantime, a significant increase in the serum HDL-C levels was observed in all AAJ-treated and positive control groups compared with that in the HFD group (Figure 1D). No statistically significant differences were found between the H-AAJ group and the SG-AAJ group regarding TC, TG, LDL-C, and HDL-C, respectively.

The activities of several enzymes including ChE, TBIL, ALT, and AST in the serum were usually used to detect early liver damage. Significant increases in ChE, TBIL, ALT, and AST activities were observed in the HFD groups compared with those in the control group, suggesting that the liver damage was induced by the HFD; however, these elevated indexes were dramatically suppressed in all AAJ-treated groups. For example, after providing AAJ-PD for 2 months, the ALT activities achieved 5.08 ± 2.20, 4.88 ± 2.79, and 4.80 ± 2.68 U/L in the L-AAJ, M-AAJ, and H-AAJ groups, respectively, with 4.57 ± 2.55 U/L in the SG-AAJ group. They were comparable with that in the positive control group (4.91 ± 3.47 U/L) but significantly lower (*p* < 0.05) than that in the HFD group (10.08 ± 5.30 U/L). Similar trends were also found in the other indices, which are shown in Figure 1E–H. No statistically significant differences were found between the H-AAJ and SG-AAJ group regarding ChE, TBIL, ALT, and AST, respectively.

**Figure 2:**
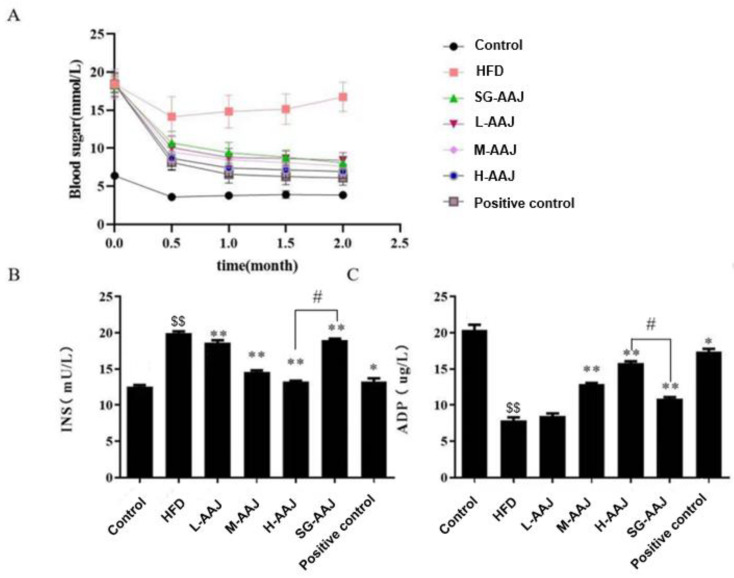
Effects of AAJ interventions on blood sugar-related indicators. (**A**) The levels of blood sugar (mmol/L), (**B**) INS (mU/L), and (**C**) ADP (μg/L). The results are expressed as mean ± standard deviation (SD, *n* = 10) shown in column graphs with error bars. Statistical analysis was performed using ANOVA. The different superscripts indicate significant difference. ^$$^ *p* < 0.01, HFD vs. control; * *p* < 0.05, ** *p* < 0.01, HFD vs. other groups; ^#^ *p* < 0.05, H-AAJ group vs. SG-AAJ group.

Significantly increased (*p* < 0.01) blood glucose levels were monitored in the HFD group compared with those in the control group; however, significantly decreased (*p* < 0.01) sugar levels were found in all the AAJ treated groups after providing AAJ product for 2 weeks compared with that in the HFD group. This trend in sugar maintained until the end of the experiment (Figure 2A). Similarly, the level of blood insulin (INS) in all AAJ-treated groups was significantly lower (*p* < 0.01) than that in the HFD group at the end of the experiment (Figure 2B). Accordingly, a significantly decreased level of adiponectin (ADP), was found in the HFD group compared with that in the control group, and the level of ADP in all the AAJ treated groups was significantly increased (*p* < 0.01) compared with that in the HFD group (Figure 2C). For example, the levels of blood sugar, INS, and ADP in the H-AAJ group after 2 months were 6.90 ± 0.9 mmol/L, 13.23 ± 0.49 mU/L, and 15.82 ± 0.76 μg/L, respectively; whereas their counterparts in the HFD group at the same time were monitored at levels of 16.80 ± 1.90 mmol/L, 19.92 ± 0.97 mU/L, and 7.88 ± 1.34 μg/L, respectively. It was found that the blood sugar levels in the SG-AAJ group and the H-AAJ group were comparable; however, the INS level in the H-AAJ group was significantly lower than that in the SG-AAJ group (*p* < 0.05), whereas the ADP level in the H-AAH group was significantly higher than that in SG-AAJ group (*p* < 0.05).

### 3.4. Indexes of the Liver, White Fat, and Brown Fat

**Figure 3:**
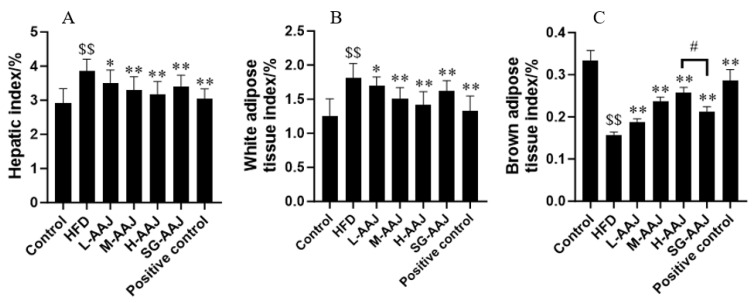
Effects of AAJ interventions on hepatic indexes (%) (**A**), WAT index (%) (**B**), and BAT index (%) (**C**). The results are expressed as mean ± standard deviation (SD, *n* = 10). Statistical analysis was performed using ANOVA. The different superscripts indicate significant differences. ^$$^ *p* < 0.01, HFD vs. control; * *p* < 0.05, ** *p* < 0.01; HFD vs. other groups; ^#^ *p* < 0.05, H-AAJ group vs. SG-AAJ group.

At the end of the experiment, the hepatic index of the rats in the HFD group (3.86 ± 0.35%) presented a significant increase after the HFD treatment when compared with that in the control group (2.92 ± 0.41%, *p* < 0.01), reflecting the remarkable liver disorder that occurred in the rats (Figure 3A). In comparison, the hepatic indexes of rats in the L-AAJ, M-AAJ, H-AAJ, and SG-AAJ groups decreased to 3.51 ± 0.37%, 3.31 ± 0.38%, 3.18 ± 0.37%, and 3.39 ± 0.34%, respectively, which were significantly lower than that in the HFD group (*p* < 0.05 for L-AAJ group, and *p* < 0.01 for the M-AAJ, H-AAJ, and SG-AAJ groups). The WAT index (Figure 3B) of the rats in the HFD group (7.84 ± 1.37%) was also significantly higher (*p* < 0.01) than that in the control group (4.14 ± 1.05%), whereas significantly lower WAT indices were monitored in the L-AAJ (6.68 ± 0.62%, *p* < 0.05), M-AAJ (5.50 ± 0.78%, *p* < 0.01), H-AAJ (5.09 ± 0.97%, *p* < 0.01), and SG-AAJ (6.12 ± 0.77%, *p* < 0.01) groups compared with that in the HFD group. Conversely, BAT indices (Figure 3C) in all AAJ-treated groups were significantly higher than that in the HFD group (*p* < 0.01 for all AAJ-treated groups). There were no statistically significant differences between the H-AAJ group and the SG-AAJ group regarding the hepatic index and WAT index, respectively, but the BWT index in the SG-AAJ group was significantly lower than that in the H-AAJ group (*p* < 0.05).

### 3.5. Pathological Analysis of Liver and Fat

**Figure 4:**
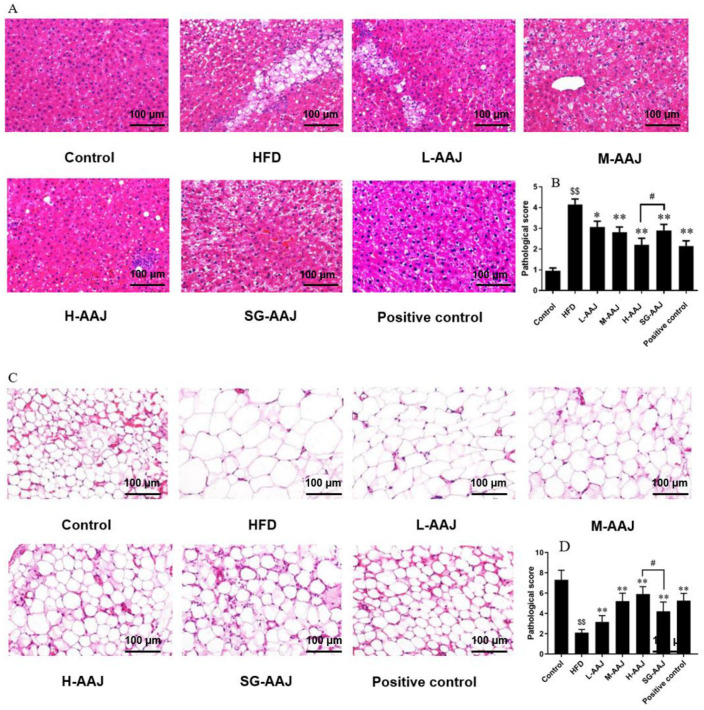

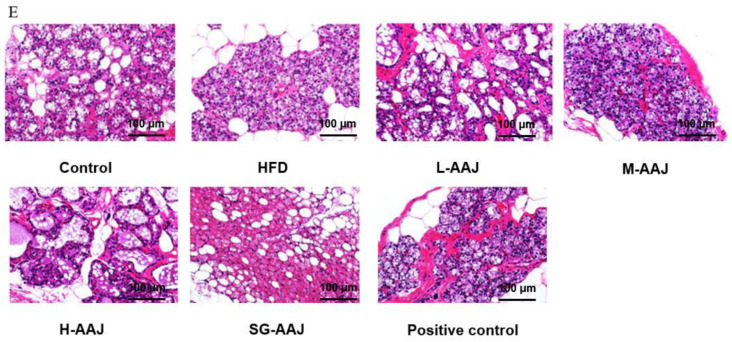
Pathological analysis of liver and fat. (**A**) HE staining of rat liver (×200). (**B**) Pathological scores of the liver. (**C**) WAT staining (×200). (**D**) Pathological scores of the WAT. (**E**) BAT staining (×200). The results are expressed as mean ± standard deviation (SD, *n* = 10). ^$$^ *p*< 0.01, HFD vs. control; * *p* < 0.05, ** *p* < 0.01, HFD vs. other groups; ^#^ *p*< 0.05, H-AAJ group vs. SG-AAJ group.

Hematoxylin–eosin (HE) staining (Figure 4) makes the nucleus appear blue, while the cytoplasm and extracellular matrix appear red. The hepatic histological results (Figure 4A) showed that the hepatic cells in the control group were regularly arranged without symptoms of fat accumulation. There were clear boundaries between cells from this group of rats, with complete nuclei mostly located in the middle of cells. In comparison, the liver cells of the HFD group were arranged in a disorderly manner, and the boundaries between the cells in this group were not as clear as that in the control group. Moreover, the HFD group showed that a large number of fat vacuoles appeared in the hepatic cells, some of which were merged to form balloon-like degeneration. Sporadic cell nodules were also seen in the livers in the HFD group. All of these findings suggested that the livers of rats in the HFD group suffered from fatty liver symptoms. Nevertheless, the lipid droplets in the AAJ-treated groups and the positive control group were significantly smaller than that of the HFD group, and the liver cells of rats in the AAJ-treated and the positive control group were more neatly arranged than those in the HFD group, suggesting that hepatic lipid accumulation was alleviated in these treatment groups. It is worth of noting that the pathological degeneration of the hepatic cells was remitted to a different extent after providing AAJ products made by using PD technology. The number of rats with severe hepatocyte vacuolar degeneration in the L-AAJ, M-AAJ, and H-AAJ groups were 7, 5, and 3, respectively. Accordingly, the number of rats with low-extent hepatocyte vacuolar degeneration in the L-AAJ, M-AAJ, and H-AAJ groups were 3, 5, and 7, respectively. Meanwhile, the results clearly suggest that these AAJ products could effectively alleviate liver damage induced by the HFD in a dose-dependent manner (Figure 4B).

For the investigation of white adipose tissues, the white fat cell compactness and the size of the fat droplet were different among the groups. The proportion of larger adipocytes and the average size of adipocytes were significantly decreased in the positive control group and all AAJ-treated groups, compared with that in the HFD group (*p* < 0.05). The fat droplets in the positive control and AAJ-treated groups also presented increased cell compactness (Figure 4C). In general, the H-AAJ group presented the strongest ability to reduce the fat accumulation, ascribed to the smallest amount of fat in the cells and the best regularity of cell compactness, followed by the positive control, M-AAJ, SG-AAJ, and L-AAJ groups (Figure 4D). The BAT in each group appeared to indicate lipid accumulation, but there was no significant difference among the groups (Figure 4E).

### 3.6. Antioxidative Indicators in Liver

**Figure 5:**
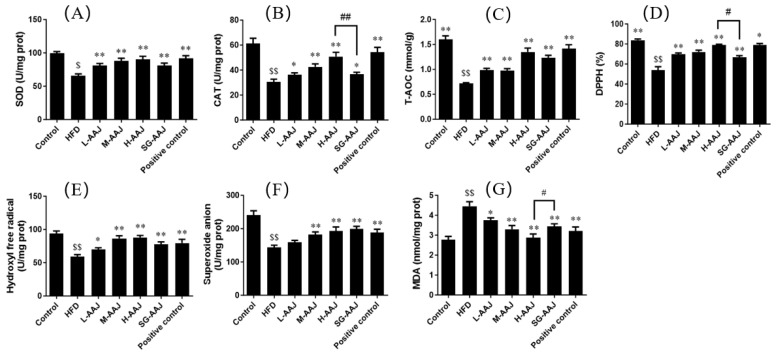
Antioxidative indicators in the liver. (**A**) SOD (U/mg protein), (**B**) CAT (U/mg protein), (**C**) T-AOC (mmol/g), (**D**), DPPH (%), (**E**) hydroxyl free radical (U/mg protein), (**F**) superoxide anion (U/mg protein), (**G**) MDA (nmol/mg protein). The results are expressed as mean ± standard deviation (SD, *n* = 10). Statistical analysis was performed using ANOVA. The means with different superscripts were considered significantly different. ^$^ *p* < 0.05, HFD vs. control; ^$$^ *p* < 0.01; * *p* < 0.05, ** *p* < 0.01, HFD vs. other groups; ^#^ *p* < 0.05, ^##^ *p* < 0.01, H-AAJ group vs. SG-AAJ group.

Compared with the control group, the HFD group presented significantly lower levels (*p* < 0.01) of SOD, CAT, T-AOC, anti-superoxide radical capacity, hydroxyl radical inhibition capacity, and DPPH antioxidant capacity. Moreover, a significantly higher level (*p* < 0.01) of MDA was observed, indicating that serious oxidative damage had occurred in the liver. Compared with the HFD group, all AAJ-treated groups presented significantly higher levels of SOD, CAT, T-AOC, anti-superoxide radical capacity, hydroxyl radical inhibition capacity, and DPPH antioxidant capacity, as well as significantly lower levels of MDA, suggesting that all AAJ products could improve the liver anti-oxidant capacity of dyslipidemia rats (Figure 5). It was also found that the CAT level in the H-AAJ group was significantly higher than that in SG-AAJ group (*p* < 0.01), as was DPPH (*p* < 0.05).

### 3.7. Transcriptome Sequencing

**Figure 6:**
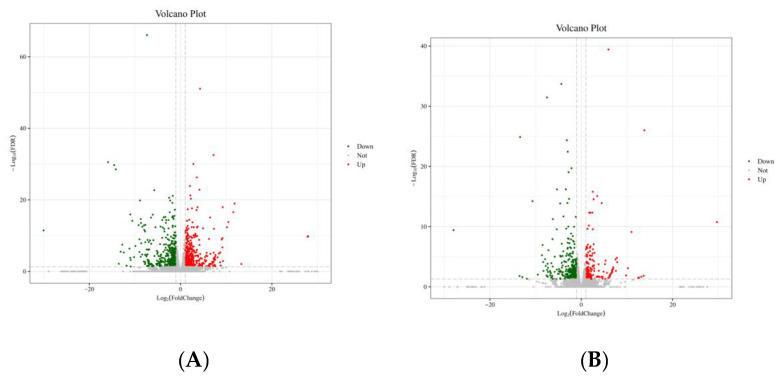

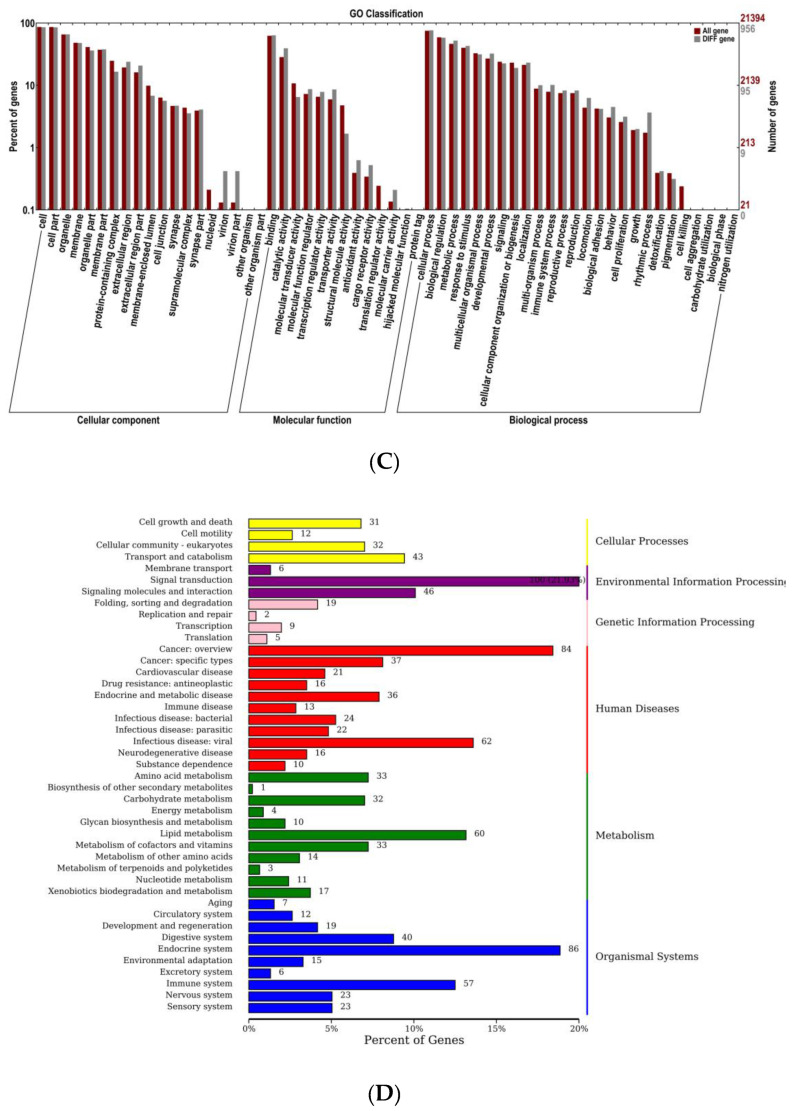

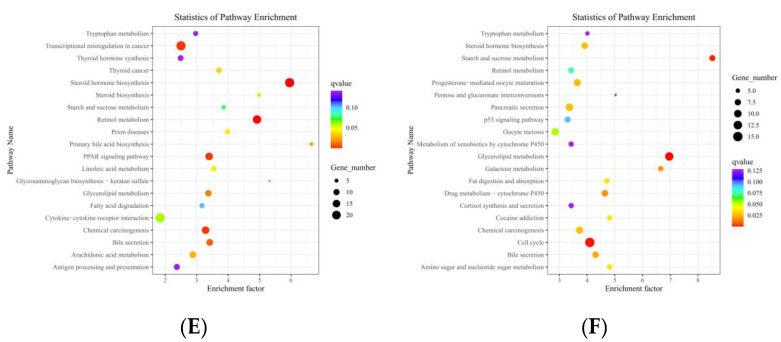
Major biological processes and cellular components of differential genes and enrichment of differentially expressed gene in KEGG pathways. (**A**,**B**) Volcano map of DEGs distribution trend among control and HFD group (**A**), and H-AAJ and HFD group (**B**). (**C**) Categorization of differentially expressed genes among HFD and control group by the GO biological processes, cellular components and molecular functions. (**D**) KEGG pathways with significant enrichment of differentially expressed genes among HFD and control groups. (**E**,**F**) KEGG classification of the control and HFD group (**E**), and H-AAJ and HFD group (**F**). All differentially expressed genes were categorized according to their GO annotations. The GO terms of biological processes, cellular components, and molecular functions are shown on *X*-axis, and the percentages and numbers of genes are shown on the left-hand side and right-hand side *Y*-axis, respectively. Differentially expressed genes identified by RNA sequencing in different groups of rat liver tissues were subjected to categorization by KEGG pathways. The numbers of differentially expressed genes are shown on the right-hand side of each bar, and the percentages of annotated genes calculated by comparison with the total gene numbers of each pathway are shown on the *X*-axis.

To learn more about AAJ’s mechanism in anti-dyslipidemia, the KEGG database, and pathway analysis enrichment was performed. The DIFFs were categorized by gene GO annotation in terms of biological processes, cellular components, and molecular functions to gain a better understanding of the major biological events involved in the therapeutic effects of AAJ on dyslipidemia. We evaluate the levels of gene expression and the expression patterns of differential genes. When related to the normal control group, 559 genes were up-regulated while 532 genes were down-regulated in the model group. Compared to the model group, 212 genes were up-regulated while 326 genes were down-regulated in the H-AAJ group. (Figure 6A,B). We discovered that the majority of the DIFFs between the control and HFD groups were associated with cellular processes, biological regulation, and metabolic processes. Red indicates the representation in the whole genome and grey indicates the representation in the DEGs. Significant differences between the distributions of GO terms for the DEGs compared to the whole genome were used to reveal the functional significance of the changes observed. For cellular components, the DIFFs were distributed into multiple subcellular components such as membrane, macromolecular complex, extracellular region, and membrane, suggesting the possible prevalent regulating mechanism of in various rat subcellular organelles. Molecular functions such as antioxidant activity, transcription regulator activity, and molecular transducer activity were involved in the HFD and control groups, as DIFFs (Figure 6C). To characterize the major biological processes and signaling transduction pathways, the DIFFs in the HFD group and the control group were significantly enriched in the lipid metabolism pathways (Figure 6D), especially steroid hormone biosynthesis PPAR signaling pathway and retinol metabolism, etc. By analysis of DIFFs, starch and sucrose metabolism and glycerolipid metabolism—were proposed among the H-AAJ and HFD groups (Figure 6E,F).

### 3.8. Effects of AAJ on Lipid Metabolism-Related Gene Transcriptions and Protein Expressions

**Figure 7:**
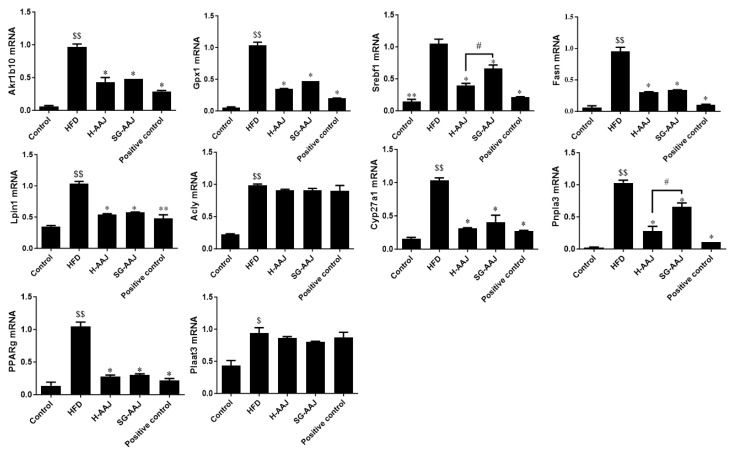
Regulation of mRNA levels of biomarkers for fatty acid synthesis and metabolism in the obese rat livers. The results are expressed as mean ± standard deviation (*n* = 10) shown in column graphs with error bars. Statistical analysis was performed using ANOVA. The means with different superscripts were considered significantly different. ^$^ *p* < 0.05, ^$$^ *p* < 0.01, HFD vs. control; * *p* < 0.05, ** *p* < 0.01, HFD vs. other groups; ^#^ *p* < 0.05 H-AAJ vs. SG-AAJ group.

**Figure 8:**
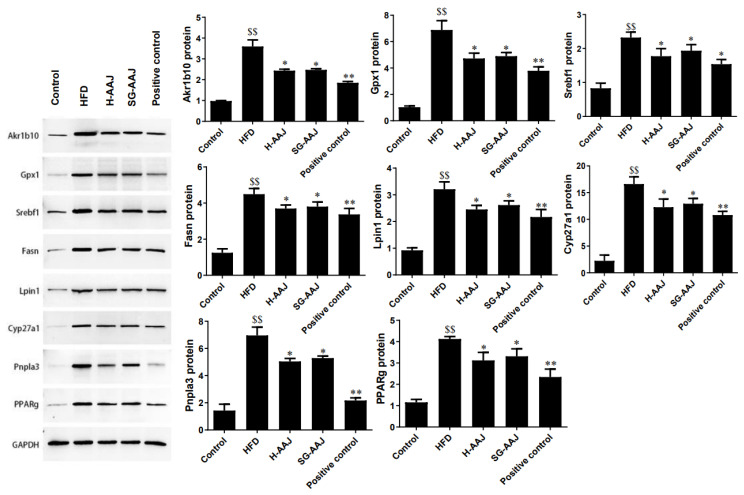
Regulation of protein levels of biomarkers for fatty acid synthesis and metabolism in the obese rat livers. The results are expressed as mean ± standard deviation (*n* = 10) shown in column graphs with error bars. Statistical analysis was performed using ANOVA. Different superscripts mean significant differences. ^$$^ *p* < 0.01, HFD vs. control; * *p* < 0.05, ** *p* < 0.01, HFD vs. other groups.

RT-PCR was used to detect the effects of AAJ products on regulating the transcription levels of lipid metabolism-related genes Aldo-keto reductase family 1 member B10 (Akr1b10), glutathione peroxidase 1 (Gpx1), sterol regulatory element-binding protein l (Srebfl), fatty acid synthase (Fasn), phosphatidate phosphatase-1 (Lpin1), ATP citrate lyase (Acly), cytochrome P450 family 27 subfamily A member 1 (Cyp27a1), patatin-like phospholipase domain-containing 3 (Pnpla3), peroxisome proliferator-activated receptor gamma (PPARg), and phospholipase A and acyltransferase 3 (Plaat3). The results were shown in Figure 7. Compared with the HFD group, Akr1b10, Gpx1, Srebfl, Fasn, Lpin1, Cyp27a1, Pnpla3, and PPARg were significantly down-regulated in the positive control, H-AAJ, and SG-AAJ groups (*p* < 0.05 or *p* < 0.01), but Acly and Plaat3 did not change significantly. It is possible that AAJ management of dyslipidemia might not be through the regulation of Acly and Plaat3, as the tested gene transcriptions did not change significantly in the AAJ-treated groups. In the second step, Western blot was used to detect the effect of AAJ products on regulating the expression levels of lipid metabolism-related proteins Akr1b10, Gpx1, Srebfl, Fasn, Lpin1, Cyp27a1, Pnpla3, and PPARg. The results were shown in Figure 8. Compared with the HFD group, Akr1b10, Gpx1, Srebfl, Fasn, Lpin1, Cyp27a1, Pnpla3, and PPARg protein in the positive control, H-AAJ, and SG-AAJ group were significantly down-regulated (*p* < 0.05 or *p* < 0.01). Acly and Plaat3 proteins were not analyzed in this section due to the similar levels of gene transcriptions between the corresponding HFD group and AAJ-treated groups.

## 4. Discussion

Current research on the functionality (i.e., regulation of lipid metabolism) of AAJ is mainly based on the polysaccharides, which are considered promising functional food supplements or therapeutic agents, showing outstanding roles in reducing blood sugar and lipids, enhancing immunity, preventing tumors, lowering alcohol, reducing blood clots, and relieving arteriosclerosis [11]. However, AAJ polysaccharides are commonly sold in their impure form since the purification of AAJ polysaccharides is relatively complicated and expensive. Polysaccharides, especially dietary fiber, have been proved to reduce the lipid accumulation in blood and liver [10,11,12]. Different processing methods may result in different physical and chemical compositions of dietary fiber and, therefore, may affect its efficacies. This work tried to find the different lipid-lowering efficacies of two AAJ products which were obtained via the super grinding method and enzymatic pre-digestion method, respectively. According to previous reports, the effective intervention dose of AAJ polysaccharides is usually 100–400 mg/kg/day in rats [1]. However, the bioactive ingredients in AAJ are not only polysaccharides, but also functional proteins/peptides, dietary fiber, polyphenols, flavonoids, and amino acids, etc. They play a synergistic role in the regulation of glucose and lipid metabolism. For example, studies have demonstrated that many flavonoids presented clear anti-dyslipidemia effects [8,23]. Therefore, it is worth noting that our research uses the whole nutritional products of AAJ after the corresponding processing technologies, i.e., the enzymatic PD treatment and the SG treatment. After analysis, the contents of polysaccharides in these two AAJ products were 0.28% and 5.12%, which means that the intervention doses of polysaccharides in the L-AAJ, M-AAJ, H-AAJ, and SG-AAJ groups were 22.68, 45.36, 90.72 mg, and 64.51 mg of polysaccharides/kg/day, which is slightly lower than the oral dose of fungus polysaccharides in the above-mentioned literature. Nevertheless, both products obtained from PD and SG technologies contained appreciable amounts of flavonoids and fibers, which could theoretically enhance the anti-dyslipidemia effects of AAJ polysaccharides in rats. Some related studies also provide support for the above insights. For example, it was found that AAJ can inhibit the weight gain in rats caused by HFD, reduce serum cholesterol, reduce liver fat accumulation, and increase Bacteroidetes. The differences in these interventions may be related to other dietary fibers present in the fungus, such as chitin, pectin, and cellulose [1].

Our study shows that both products obtained from PD and SG technologies alleviate lipid levels to some extent, thereby possibly promoting lipid metabolism. In this study, we have shown that the effect of the AAJ on attenuating the increase in serum TC and LDL-C is comparable with that of Jiaogulan tea (positive control group), with the latter containing a family of naturally occurring statins [24]. Particularly, the ratios of TC/HDL-C and LDL-C/HDL-C have been used as CVD risk factors [25], and AAJ treatment ameliorated these factors. Moreover, the serum TC levels are considered as a key risk indicator of arterial endothelial dysfunction [26]. The elevated TC levels may result in the progression of atherosclerosis. AAJ administration significantly alleviates high-fat diet-induced dyslipidemia by decreasing the serum levels of TG, TC, and LDL-C. It is logical to speculate that AAJ may promote the transportation of TC and TG from plasma to the liver by increasing the serum HDL-C level and reducing the serum LDL-C level [27].

The liver is an essential organ for lipid metabolism and plays an important role in the biosynthesis and β-oxidation of free fatty acids. Lipid drops are normally accumulated in hepatic tissue and induce structural and functional changes in the liver, eventually causing dyslipidemia [28]. Therefore, reducing fat accumulation in the liver can prevent the development of lipid metabolic disorders. In this study, it was shown that HFD-induced hepatic lipid accumulation was evidently observed with abnormal hepatic indexes (Figure 3). Furthermore, increased hepatic fat accumulation was observed; therefore, it was hypothesized that the fatty liver occurred in this group. In comparison, the use of AAJ presented remarkable improvement in reversing the symptom of fatty liver. Particularly, reduced fatty droplets were shown in the hepatocytes with the increase in the dose of AAJ products made by using the PD technique, demonstrating appreciable hepatic-protective ability by restoring normal functions of fatty liver in dyslipidemia rats, though the potencies were different. The H-AAJ group clearly presented a lower pathological score than that of the SG group. There are two major types of adipose tissues, namely the WAT and BAT. The WAT is the predominant type of fat in the body that serves as an energy-storing reservoir; on the other hand, the BAT is used to generate heat through non-shivering thermogenesis—a process that is especially important to preventing hypothermia [29]. In obesity, it is commonly observed that adipose tissues are increasingly accumulated, and cellular hypertrophy appeared to be the major mode of expansion in the intra-abdominal adipose tissue in rodents [30]. All AAJ products clearly suppressed the expansion of fat tissues in dyslipidemia rats. Adipose tissue also serves as an endocrine and secretory organ [31]. Adipose tissues secret adipokines, and the changes in the adipokines’ profiles in obesity are possibly implicated in the development of many diseases, including insulin resistance [32]. ADP, an adipokine with unique metabolic properties, was increased by all the AAJ treatments. Given the anti-inflammatory properties of ADP [33], it was logical to speculate that AAJ products had beneficial effects on cardiovascular and metabolic disorders including atherosclerosis and insulin resistance.

Oxidative stress injuries both in the blood and liver are the result of lipid accumulation toxicity. Liver dysfunction resulting from liver injury and hepatic fat accumulation may apparently lead to abnormal lipid metabolism. Previous studies have shown that elevated AST and ALT could be used as biomarkers of liver damage [23]. It was also found that the AST and ALT levels of the rats receiving HFD were significantly higher than those in the control group when investigating HFD-induced non-alcoholic fatty liver disease [34]. In this study, it was shown that AAJ intervention could alleviate liver injury and hepatic fat accumulation to a certain degree, thus potentially promoting lipid metabolism. It had been widely accepted that enhanced oxidative stress might induce tissue damage; therefore, one of the possible mechanisms of AAJ to relieve liver damage was to prevent the buildup of oxidative stress by restoring the anti-oxidant defense system—all the AAJ treated groups presented significantly higher levels of SOD, CAT, T-AOC, anti-superoxide radical capacity, hydroxyl radical inhibition capacity, and DPPH antioxidant capacity. In addition, liver MDA values, an index of lipid peroxidation that can be used to reflect the extent of liver injury, were effectively reduced. It was noteworthy that the H-AAJ group presented stronger effects in promoting the levels of CAT and DPPH while reducing the levels of MDA than the SG-AAJ group, demonstrating that the higher amounts of flavonoids released by PD technology were possibly indeed advantageous in maintaining an antioxidant environment in vivo.

Gynostemma (Jiaogulan Tea), a blood lipid-regulating health food with a single raw material approved for sale in China as a food supplement, was selected as the positive control in this experiment. Our results showed that the H-AAJ group and positive control group presented almost equal pharmacological effects in many aspects, for example, in reducing sugar and INS levels (Figure 2), and in reducing pathological scores in the liver (Figure 4). This means the use of a high dose of AAJ made by using PD technology (H-AAJ group) could be a good alternative to the Jiaogulan Tea.

Large numbers of differently expressed genes were identified in dyslipidemia rats treated with AAJ products in the AAJ-treated groups by sequencing technology. By GO and KEGG annotation, these differently expressed genes could be grouped into various biological processes and signaling pathways, including glycolipid metabolism and inflammatory factors, showing the versatile functions of AAJ in regulating cellular functions. In this study, by analysis of transcriptome sequencing among the AAJ-treated rats, a KEGG pathway—starch and sucrose metabolism and glycerolipid metabolism—was proposed, and the appreciable ability of AAJ in modulating normal functions and regulating dyslipidemia disorders via these signaling pathways was also shown, which provided the fundament to elucidating the mechanism. Through the transcriptome sequencing data, we selected the following 10 genes with significant differences related to the glyceride metabolism pathway.

As demonstrated in the study, the accumulation of hepatic triglycerides is a manifestation in the HFD group. In the M-AAJ group, the imbalance in liver lipid metabolism led to the accumulation of triglycerides and steatosis. Hepatic lipid synthesis and metabolism are regulated by gene and protein expressions of factors related to fatty acid synthesis, uptake, and transport. To elucidate the mechanisms of AAJ in regulating lipid levels, the study investigated lipid synthesis and metabolism-related gene mRNA and protein expressions in the hepatic tissues. Several key biomarkers, including Akr1b10, Gpx1, Srebfl, Fasn, Lpin1, Acly, Cyp27a1, Pnpla3, PPARg, and Plaat3, were subjected to analysis, and the changes in these differentially expressed genes (DEGs) can preliminarily explain the mechanism of black fungus intervention in regulating lipid metabolism. Among these factors; AKR1B10 is a novel regulator of fatty acid de novo synthesis [35]; Gpx1 is related to fatty acid oxidation [36]; SREBPs regulate the expression of genes required for the synthesis of fatty acids and cholesterol [37]; Fasn plays a key role in lipogenesis [38]; Lpin1 regulates fatty acid oxidation [39]; Acly is a cytosolic enzyme that catalyzes the generation of acetyl-CoA, which is a vital building block for fatty acid [40]; CYP27A1 is a key enzyme in bile acids biosynthesis and a regulator of cholesterol metabolism [41]; Pnpla 3 has also been suggested to play a role in lipid remodeling in hepatic TG [42]; PPARs are responsible for fatty acids metabolism [43]; and Plaat3 is also related to lipid metabolism [44]. The results clearly show that AAJ could significantly down-regulate most of these factors in the rat liver. Therefore, our study strongly suggests that AAJ shows lipid-lowering effects through its effects on lipid metabolism in the liver.

## 5. Conclusions

In conclusion, the present study suggests that AAJ intervention could result in the loss of body weight and fat tissue weight in HFD-fed rats, and this effect is dose-dependent. Smaller liver index and fat index were also recorded. Ameliorated serum lipid status and alleviated fatty liver symptoms were achieved by continuous oral administration of AAJ for an additional term. AAJ treatment highly activated anti-oxidation processes. Upregulation of adiponectin from AAJ treatment was also found. The improvement effects of AAJ on the rats were observed by assessing key parameters for anti-obesity and anti-diabetic activities. Deep-processed AAJ created by using PD technology appears to have better performance in regulating glucose and lipid metabolism than SG product, which should be attributed to the higher levels of dietary fiber, crude polysaccharides, and total flavonoids residing in PD product. Furthermore, by analysis of transcriptome sequencing among the AAJ-treated rats, a KEGG pathway—starch and sucrose metabolism and glycerolipid metabolism—was proposed. The study also showed that AAJ lowered hepatic triglyceride levels through the regulation of hepatic fatty acid synthesis and metabolism in rats. Collectively, AAJ products have the effect of anti-obesity, regulating blood lipids, and alleviating fatty liver. Its mechanism of action is related to regulating the expression of genes and proteins related to lipid synthesis and metabolism. Moreover, these findings illustrated that AAJ product made by PD technology possibly had the greatest potential to improve anti-oxidative performance and ameliorate lipid metabolism disorder; therefore, it could be regarded as an attractive potential functional food for the management of dyslipidemia.

## Figures and Tables

**Table 1:** The primer sequences used in the real-time reverse transcription PCR analysis.

Gene Symbol	Forward Primer (5′→3′)	Reverse Primer (5′→3′)	Length (bps)
**GAPDH**	5′-CCTCTGACTTCAACAGCGACAC-3′	5′-CTGTTGCTGTAGCCAAATTCGT-3′	121
**Akr1b10**	5′-CAAGTCTGTGACACCCTCCC-3′	5′-GCCCTCCAGTTCCTGTTGAA-3′	108
**Gpx1**	5′-CCGGGACTACACCGAAATGA-3′	5′-TGCCATTCTCCTGATGTCCG-3′	104
**Srebf1**	5′-CGTTTCTTCGTGGATGGGGA-3′	5′-ACAGTTCAATGCTCGCTCCA-3′	141
**Fasn**	5′-GCTTGGTGAACTGTCTCCGA-3′	5′-GTGAGATGTGCTGCTGAGGT-3′	83
**Acly**	5′-CAGTGGGAGCACATCGACAC-3′	5′-ACTCTTTCCTTGGGGCATGG-3′	115
**Cyp27a1**	5′-GCTCCAGGCGCTGAACAAG-3′	5′-CACTGCTCCATGCTGTCTCT-3′	150
**Pnpla3**	5′-GGGGCTACGCTATGTCTGAG-3′	5′-GATCGAGAGGGAGACTGCAC-3′	121
**Pparg**	5′-TCCCGTTCACAAGAGCTGAC-3′	5′-ATAATAAGGCGGGGACGCAG-3′	107
**Plaat3**	5′-TGGCTCCCCCAAGTGAAATC-3′	5′-GGTACTTGTCCTTCCCAGCC-3′	117
**Lpin1**	5′-AGCTCCTCTACTTCTGGCGA-3′	5′-TGTGGCGTTTCTTCCTCTCC-3′	133

**Table 2:** Analysis of basic components.

	Total Protein (g/100 g)	Dietary Fiber (g/100 g)	Crude Polysaccharides (g/100 g)	Total Flavonoids (mg/100 g)
PD product of AAJ	0.53	1.78	0.28	410
SG product of AAJ	0.52	1.10	0.20	8.4

**Table 3:** Effect of AAJ interventions on body weight (mean ± SD).

Groups	Control	HFD	SG-AAJ	L-AAJ	M-AAJ	H-AAJ	Positive Control
0 Week	193.8 ± 11.4	192.7 ± 6.5	194.3 ± 8.4	191.5 ± 8.1	190.4 ± 10.1	191.9 ± 9.7	189.4 ± 6.0
1 Week	204.8 ± 12.9	214.5 ± 6.7	210.7 ± 10.2	209.9 ± 9.8	206.3 ± 9.4	210.2 ± 9.8	209.4 ± 6.7
2 Week	218.3 ± 12.6	233.5 ± 8.2 ^$^	231.3 ± 10.2	230.0 ± 12.4	227.1 ± 12.1	231.8 ± 8.7	231.2 ± 7.6
3 Week	229.6 ± 12.8	254.8 ± 9.6 ^$^	249.3 ± 11.7	253.5 ± 13.4	248.2 ± 14.3	255.7 ± 10.4	250.2 ± 10.2
4 Week	243.9 ± 14.0	281.0 ± 11.3 ^$$^	266.6 ± 12.8 *	273.5 ± 16.3	263.2 ± 15.8 *	268.4 ± 11.0 *	263.4 ± 10.1 **
5 Week	254.7 ± 15.7	304.6 ± 11.9 ^$$^	280.9 ± 14.1 **	292.1 ± 17.1	275.7 ± 14.9 **	281.3 ± 12.3 **	274.6 ± 10.8 **
6 Week	267.6 ± 17.1	322.3 ± 13.8 ^$$^	298.2 ± 15.6 **	307.7 ± 18.0	291.4 ± 15.3 **	294.7 ± 13.3 **	286.7 ± 13.1 **
7 Week	277.5 ± 17.8	342.3 ± 15.2 ^$$^	310.6 ± 16.5 **	325.2 ± 18.2 *	306.3 ± 17.3 **	307.9 ± 13.8 **	300.8 ± 14.4 **
8 Week	286.3 ± 18.6	360.5 ± 16.4 ^$$^	324.9 ± 18.1 **	339.8 ± 18.9 *	317.6 ± 17.5 **	315.8 ± 16.1 **	310.1 ± 17.2 **
9 Week	296.6 ± 20.2	381.9 ± 20.0 ^$$^	338.7 ± 19.6 **	352.2 ± 23.1 **	331.6 ± 20.7 **	327.8 ± 17.6 **	320.6 ± 17.4 **
10 Week	308.7 ± 21.2	400.2 ± 22.0 ^$$^	351.9 ± 21.2 **	368.1 ± 22.5 **	344.1 ± 21.1 **	338.6 ± 19.4 **	328.7 ± 17.6 **

Groups: HFD, high fat diet group; SG-AAJ, superfine grinding of AAJ (6.3 g/kg) group; L-AAJ, low dose (28.8 mL/kg); M-AAJ, middle dose (57.6 mL/kg); H-AAJ, high dose (115.2 mL/kg) of AAJ with pre-digestion technique; positive control group (Jiaogulan dose of 0.72 g/kg). The results are expressed as mean ± standard deviation (SD, *n* = 10). One-way ANOVA method was used for statistical analyses. ^$^ *p* < 0.05, ^$$^ *p* < 0.01, HFD vs. control; * *p* < 0.05, ** *p* < 0.01, compared to HFD.

## Data Availability

Data is contained within the article.

## Abbreviations

AAJ, *Auricularia auricula-judae*; Acly, ATP citrate lyase; Akr1b10, Aldo-keto reductase family 1 member B10; ALT, alanine aminotransferase; ADP, adiponectin; AST, aspartate aminotransferase; BAT, brown adipose tissue; CAT, catalase; ChE, cholinesterase; CVD, cardiovascular disease; Cyp27a1, cytochrome P450 family 27 subfamily A member 1; Fasn, fatty acid synthase; Gpx1, glutathione Peroxidase 1; HDL-C, high-density lipoprotein cholesterol; HE, hematoxylin and eosin; HFD, high-fat diet; HI, hepatic index; INS, insulin; LDL-C, low-density lipoprotein cholesterol; Lpin1, phosphatidate phosphatase-1; MDA, malondialdehyde; PCR, polymerase chain reaction; PD, pre-digestion; Plaat3, phospholipase A and acyltransferase 3; Pnpla3, patatin-like phospholipase domain-containing 3; PPARg, peroxisome proliferator activated receptor gamma; SOD, Superoxide dismutase; SG, superfine grinding; Srebfl, sterol regulatory element-binding protein l; TBIL, total bilirubin; TC, total cholesterol; TG, triglyceride; T-AOC, total antioxidant capacity; WAT, white adipose tissue.

## References

[1] Zhang T., Zhao W., Xie B., Liu H. (2020). Effects of *Auricularia auricula* and its polysaccharide on diet-induced hyperlipidemia rats by modulating gut microbiota. Foods Funct..

[2] World Health Organization (WHO) Data: Raised Cholesterol. https://www.who.int/data/gho/indicator-metadata-registry/imr-details/3236.

[3] Sirtori C.R., Galli C., Anderson J.W., Arnoldi A. (2009). Nutritional and nutraceutical approaches to dyslipidemia and atherosclerosis prevention: Focus on dietary proteins. Atherosclerosis.

[4] Wang J., Li P., Liu S., Zhang B., Hu Y., Ma H., Wang S. (2020). Green tea leaf powder prevents dyslipidemia in high-fat diet-fed mice by modulating gut microbiota. Food Nutr. Res..

[5] Khalifa S.A.M., Elashal M.H., Yosri N., Du M., Musharraf S.G., Nahar L., Sarker S.D., Guo Z., Cao W., Zou X. (2021). Bee pollen: Current status and therapeutic potential. Nutrients.

[6] Baumgartner S., Mensink R.P., Plat J. (2011). Plant sterols and stanols in the treatment of dyslipidemia: New insights into targets and mechanisms related to cardiovascular risk. Curr. Pharm. Des..

[7] Mohamadshahi M., Veissi M., Haidari F., Javid A.Z., Mohammadi F., Shirbeigi E. (2014). Effects of probiotic yogurt consumption on lipid profile in type 2 diabetic patients: A randomized controlled clinical trial. J. Res. Med. Sci..

[8] Diane A., Borthwick F., Wu S., Lee J., Brown P.N., Dickinson T.A., Croft K.D., Vine D.F., Proctor S.D. (2016). Hypolipidemic and cardioprotective benefits of a novel fireberry hawthorn fruit extract in the JCR:LA-cp rodent model of dyslipidemia and cardiac dysfunction. Food Funct..

[9] Hosseini A., Hosseinzadeh H. (2015). A review on the effects of *Allium sativum* (Garlic) in metabolic syndrome. J. Endocrinol. Investig..

[10] Liu Q., Ma R., Li S., Fei Y., Lei J., Li R., Pan Y., Liu S., Wang L. (2022). Dietary supplementation of *Auricularia auricula-judae* polysaccharides alleviate nutritional obesity in mice via regulating inflammatory response and lipid metabolism. Foods.

[11] Islam T., Ganesan K., Xu B. (2021). Insights into health-promoting effects of Jew’s ear (*Auricularia auricula-judae*). Trends Food Sci. Technol..

[12] Liu E., Ji Y., Zhang F., Liu B., Meng X. (2021). Review on *Auricularia auricula-judae* as a functional food: Growth, chemical composition, and biological activities. J. Agric. Food Chem..

[13] Li G., Guo W., Gao X., Wang Y., Sun S. (2020). Effect of superfine grinding on physicochemical and antioxidant properties of soybean residue powder. Food Sci. Nutr..

[14] Gong P., Huang Z., Guo Y., Wang X., Yue S., Yang W., Chen F., Chang X., Chen L. (2022). The effect of superfine grinding on physicochemical properties of three kinds of mushroom powder. J. Food Sci..

[15] Zhang M., Wang F., Liu R., Tang X., Zhang Q., Zhang Z. (2014). Effects of superfine grinding on physicochemical and antioxidant properties of *Lycium barbarum* polysaccharides. LWT Food Sci. Technol..

[16] Tang C.H., Wang X.S., Yang X.Q. (2009). Enzymatic hydrolysis of hemp (*Cannabis sativa* L.) protein isolate by various proteases and antioxidant properties of the resulting hydrolysates. Food Chem..

[17] Rizvi N.B., Aleem S., Khan M.R., Ashraf S., Busquets R. (2022). Quantitative estimation of protein in sprouts of *Vigna radiate* (Mung Beans), *Lens culinaris* (Lentils), and *Cicer arietinum* (Chickpeas) by Kjeldahl and Lowry methods. Molecules..

[18] Merten D., Erman L., Marabelli G.P., Leners B., Ney Y., Nasim M.J., Jacob C., Tchoumtchoua J., Cajot S., Bohn T. (2022). Potential health effects of brewers’ spent grain as a functional food ingredient assessed by markers of oxidative stress and inflammation following gastro-intestinal digestion and in a cell model of the small intestine. Food Funct..

[19] Zhang H.F., Niu L.L., Yang X.H., Li L. (2014). Analysis of water-soluble polysaccharides in an edible medicinal plant Epimedium: Method development, validation, and application. J. AOAC Int..

[20] Mao H.M., Xiang X.S., Li Y., Zhao J.P., Huang Y., Di S.S., Zhuo Q., Nie H.G. (2023). Analysis of metabolite distribution in rat liver of high-fat model by mass spectrometry imaging. Metabolites.

[21] Megalli S., Davies N.M., Roufogalis B.D. (2006). Anti-hyperlipidemic and hypoglycemic effects of *Gynostemma pentaphyllum* in the Zucker fatty rat. J. Pharm. Pharm. Sci..

[22] Ren Q., Liu X.Q., Zhou X.W., Zhou X., Fang G., Wang B., Wang Y.P., Peng D.H., Li X.T. (2021). Effects of Huatan Jiangzhuo decoction on diet-induced hyperlipidemia and gene expressions in rats. Chin. J. Nat. Med..

[23] Khan T.J., Kuerban A., Razvi S.S., Mehanna M.G., Khan K.A., Almulaiky Y.Q., Faidallah H.M. (2018). In vivo evaluation of hypolipidemic and antioxidative effect of ‘Ajwa’ (*Phoenix dactylifera* L.) date seed-extract in high-fat diet-induced hyperlipidemic rat model. Biomed. Pharmacother..

[24] Wang M., Wang F., Wang Y., Ma X., Zhao M., Zhao C. (2013). Metabonomics study of the therapeutic mechanism of *Gynostemma pentaphyllum* and atorvastatin for hyperlipidemia in rats. PLoS ONE.

[25] Millán J., Pintó X., Muñoz A., Zúñiga M., Rubiés-Prat J., Pallardo L.P., Masana L., Mangas A., Hernández-Mijares A., González-Santos P. (2009). Lipoprotein ratios: Physiological significance and clinical usefulness in cardiovascular prevention. Vasc. Health Risk Manag..

[26] Steinberg H.O., Bayazeed B., Hook G., Johnson A., Cronin J., Baron A.D. (1997). Endothelial dysfunction is associated with cholesterol levels in the high normal range in humans. Circulation.

[27] Lv X.C., Guo W.L., Lia L., Yu X.D., Liu B. (2019). Polysaccharide peptides from *Ganoderma lucidum* ameliorate lipid metabolic disorders and gut microbiota dysbiosis in high-fat diet-fed rats. J. Funct. Foods..

[28] Mashek D.G. (2021). Hepatic lipid droplets: A balancing act between energy storage and metabolic dysfunction in NAFLD. Mol. Metab..

[29] Marlatt K.L., Ravussin E. (2017). Brown adipose tissue: An update on recent findings. Curr. Obes. Rep..

[30] Kim J.W., Lee Y.S., Seol D.J., Cho I.J., Ku S.K., Choi J.S., Lee H.J. (2018). Antiobesity and fatty liver-preventing activities of *Lonicera caerulea* in high-fat diet-fed mice. Int. J. Mol. Med..

[31] Fujita H., Fujishima H., Koshimura J., Hosoba M., Yoshioka N., Shimotomai T., Morii T., Narita T., Kakei M., Ito S. (2005). Effects of antidiabetic treatment with metformin and insulin on serum and adipose tissue adiponectin levels in db/db mice. Endocr. J..

[32] Kang S.J., Lee J.E., Lee E.K., Jung D.H., Song C.H., Park S.J., Kang S.J., Lee J.E., Lee E.K., Jung D.H. (2014). Fermentation with *Aquilariae lignum* enhances the anti-diabetic activity of green tea in type II diabetic db/db mouse. Nutrients.

[33] Ouchi N., Walsh K. (2007). Adiponectin as an anti-inflammatory factor. Clin. Chim. Acta.

[34] Liu Y.L., Zhang Q.Z., Wang Y.R., Fu L.N., Han J.S., Zhang J., Wang B.M. (2021). Astragaloside IV improves high-fat diet-induced hepatic steatosis in nonalcoholic fatty liver disease rats by regulating inflammatory factors level via TLR4/NF-κB signaling pathway. Front. Pharmacol..

[35] Ma J., Yan R., Zu X., Cheng J.M., Rao K., Liao D.F., Cao D. (2008). Aldo-keto reductase family 1 B10 affects fatty acid synthesis by regulating the stability of acetyl-CoA carboxylase-alpha in breast cancer cells. J. Biol. Chem..

[36] Grim J.M., Hyndman K.A., Kriska T., Girotti A.W., Crockett E.L. (2011). Relationship between oxidizable fatty acid content and level of antioxidant glutathione peroxidases in marine fish. J. Exp. Biol..

[37] Bengoechea-Alonso M.T., Ericsson J. (2007). SREBP in signal transduction: Cholesterol metabolism and beyond. Curr. Opin. Cell Biol..

[38] Flavin R., Peluso S., Nguyen P.L., Loda M. (2010). Fatty acid synthase as a potential therapeutic target in cancer. Future Oncol..

[39] Jiang L., Bi Z., Zhou J. (2015). The role of lipin-1 in the pathogenesis of alcoholic fatty liver. Alcohol Alcohol..

[40] Zaidi N., Royaux I., Swinnen J.V., Smans K. (2012). ATP citrate lyase knockdown induces growth arrest and apoptosis through different cell- and environment-dependent mechanisms. Mol. Cancer Ther..

[41] Zurkinden L., Sviridov D., Vogt B., Escher G. (2020). Downregulation of Cyp7a1 by cholic acid and chenodeoxycholic acid in Cyp27a1/ApoE double knockout mice: Differential cardiovascular outcome. Front. Endocrinol..

[42] Basu Ray S. (2019). PNPLA3-I148M: A problem of plenty in non-alcoholic fatty liver disease. Adipocyte.

[43] Varga T., Czimmerer Z., Nagy L. (2011). PPARs are a unique set of fatty acid regulated transcription factors controlling both lipid metabolism and inflammation. Biochim. Biophys. Acta.

[44] Uyama T., Tsuboi K., Ueda N. (2017). An involvement of phospholipase A/acyltransferase family proteins in peroxisome regulation and plasmalogen metabolism. FEBS Lett..

